# Development of a DC-Biased AC-Stimulated Microfluidic Device for the Electrokinetic Separation of Bacterial and Yeast Cells

**DOI:** 10.3390/bios14050237

**Published:** 2024-05-09

**Authors:** Nuzhet Nihaar Nasir Ahamed, Carlos A. Mendiola-Escobedo, Victor H. Perez-Gonzalez, Blanca H. Lapizco-Encinas

**Affiliations:** 1 Microscale Bioseparations Laboratory, Biomedical Engineering Department, Rochester Institute of Technology, 160 Lomb Memorial Drive, Rochester, NY 14623, USA; nn5878@rit.edu (N.N.N.A.); cm2379@rit.edu (C.A.M.-E.); 2 School of Engineering and Sciences, Tecnologico de Monterrey, Monterrey 64700, Nuevo Leon, Mexico

**Keywords:** electrokinetics, alternating current, insulating posts, microfluidics, microorganisms

## Abstract

Electrokinetic (EK) microsystems, which are capable of performing separations without the need for labeling analytes, are a rapidly growing area in microfluidics. The present work demonstrated three distinct binary microbial separations, computationally modeled and experimentally performed, in an insulator-based EK (iEK) system stimulated by DC-biased AC potentials. The separations had an increasing order of difficulty. First, a separation between cells of two distinct domains (*Escherichia coli* and *Saccharomyces cerevisiae*) was demonstrated. The second separation was for cells from the same domain but different species (*Bacillus subtilis* and *Bacillus cereus*). The last separation included cells from two closely related microbial strains of the same domain and the same species (two distinct *S. cerevisiae* strains). For each separation, a novel computational model, employing a continuous spatial and temporal function for predicting the particle velocity, was used to predict the retention time ($t_{R,p}$) of each cell type, which aided the experimentation. All three cases resulted in separation resolution values $R_{s}>1.5,$ indicating complete separation between the two cell species, with good reproducibility between the experimental repetitions (deviations < 6%) and good agreement (deviations < 18%) between the predicted $t_{R,p}$ and experimental ($t_{R,e}$) retention time values. This study demonstrated the potential of DC-biased AC iEK systems for performing challenging microbial separations.

## 1. Introduction

An attractive option for the rapid assessment of microbes is microscale electrokinetic (EK) devices, which possess beneficial characteristics such as low cost, portability and low sample requirements [1,2]. The separation and discrimination of microorganisms, especially bacteria and yeast, is essential in applications for food safety, clinical analysis, and environmental monitoring. Microscale EK systems have the potential to become an alternative for the rapid analysis of samples containing multiple types of cells, particularly those that are pathogenic to humans or animals and can contaminate food items and the environment [3,4,5]. Therefore, there exists an increasing demand for the development of new reliable and robust separation methods for microorganisms [6,7,8]. Conventional filtration and culture-based methods for separating microorganisms can be labor-intensive and time-consuming, thus creating a need for rapid response methods in microbial analysis [6].

One of the well-established methods for the rapid characterization and separation of intact microorganisms involves capillary electrophoresis (CE) systems employing direct current (DC) voltages [9,10,11]. These systems have been investigated by several research groups, such as the Armstrong [12,13,14], Horká [15,16,17], and Buszewski [18,19,20] groups. Traditional CE methods for microbial analysis, pioneered in the early 1980s by Hjertén [21], Jorgenson [22], and Armstrong [23], have now been extended to the separation and evaluation of bacteria [24,25,26] and bacterial aggregates [27,28,29]. Though CE-based separations are excellent for the rapid detection and separation of intact microorganisms, their applications are limited to the use of only linear EK phenomena, limiting their discriminatory capabilities [30,31,32].

Insulator-based EK (iEK) devices possess the unique capability of combining linear and nonlinear EK effects within the same system [33,34], which enables the separation of complex mixtures, including intact microbes [35,36,37,38]. The utilization of DC-stimulated iEK (DC-iEK) devices for separating microorganisms has been reported by several groups. The Hayes research group reported a separation between serotypes of *Salmonella* [39], trapping of *Listeria monocytogenes* [40], and differentiation between methicillin-resistant and methicillin-susceptible *Staphylococcus aureus* (*S. aureus*) [41]. The Buie group employed a 3D iEK system to trap *Escherichia coli* (*E. coli*) and *Bacillus cereus* (*B. cereus*) cells, and to discriminate between pathogenic strains of *Streptococcus mitis* and *Pseudomonas aeruginosa* [42]. Our group also reported the continuous separation of yeast and bacterial cells [43].

There are only a handful of reports on microbial separations performed through the application of alternating-current (AC) voltages in iEK devices, where parameters such as the frequency (*f*), peak amplitude (*V_p_*) and DC bias can be varied. The Agah group demonstrated the separation of *E. coli* from microparticles [44] and the selective trapping of live *S. aureus* cells from dead *S. aureus* cells [45] by employing DC-biased AC electric potentials with frequencies > 1 kHz for the separations. A few groups have investigated the utilization of DC-biased AC potentials with frequencies < 1 kHz for separating microorganisms. The Xuan group demonstrated this by focusing of yeast cells in a serpentine microchannel [46], and in a virtually “infinite” microchannel [47], by employing low-frequency AC voltages. Our group investigated the effects of fine-tuning DC-biased AC potentials and manipulating the insulating post array on microparticle separation [48,49] and compared the separation of *E. coli* and *Saccharomyces cerevisiae* (*S. cerevisiae*) cells using DC-biased low-frequency AC signals [50]. We reported that the application of DC-biased AC potentials had an added advantage in comparison with DC signals [50] when applied to iEK systems. Thus, the potential of these iEK systems subjected to DC-biased low-frequency AC signals was unveiled for separating more challenging cell mixtures with similar characteristics.

The present study leverages previous reports focused on fine-tuning the characteristics of the applied AC potential [48] and iEK device [49], respectively. Presented here is the application of this new knowledge for the separation of three distinct biological samples. Specifically, this work demonstrated three binary microbial separations performed in DC-biased AC-iEK systems with an increasing order of difficulty. This study comprised mathematical modeling and experimentation to design and perform the three distinct EK-based separations, involving both spherical and non-spherical cells, by applying a low-frequency DC-biased AC voltage. The same voltage sequence was employed in all the separations. Both linear and nonlinear EK phenomena were considered. The first separation demonstrated discrimination between *E. coli* and *S. cerevisiae*, which are cells from distinct domains, prokaryotic and eukaryotic, respectively. The second separation, with a higher level of difficulty, between *Bacillus subtilis* (*B. subtilis*) and *B. cereus* cells demonstrated the discrimination between cells from the same prokaryotic domain and from different species. The third separation was designed with an even higher degree of difficulty and differentiated cells from the same eukaryotic domain, same species and only different strains of *S. cerevisiae* cells. The range of cells chosen for this study contained both bacterial and yeast cells in order to test the proposed technique with distinct types of cells that are relevant in clinical, food and environmental analysis. The quality of these separations was evaluated and quantified in terms of the separation resolution (*R_s_*) by assessing the electropherograms. For all the separations, a *R_s_* ≥ 1.5 was achieved, indicating that the separations were complete. Good reproducibility, which was quantified by deviations ranging from 2.1 to 5.2% between the experimental repetitions, was obtained. Additionally, the quantitative agreement between the predicted and experimental retention times ranged from −6.4 to 17.8%, indicating that the novel computational model, employing spatial and temporal functions for the electric field intensity, is a helpful tool for designing microbial separations. This report is the first demonstration of the separation of closely related microbial strains, possessing spherical- and non-spherical-shaped cells, using low-frequency AC potentials in iEK systems, while considering both linear and nonlinear EK phenomena, including the effects of EP_NL_. This report illustrates the potential of low-frequency DC-biased AC-iEK systems to achieve highly discriminatory separations of microorganisms with similar characteristics.

## 2. Theory

Based on the dependence of the electrokinetic phenomena on the electric field, the EK phenomena are classified as linear and nonlinear. The linear electroosmotic (EO) flow and linear electrophoresis (EP_L_) are the linear EK phenomena considered here. The velocity of these phenomena depends linearly on the electric field, $E=E{\hat{a}}_{E}$ (where ${\hat{a}}_{E}$ is a unit vector with the direction of vector E, having a magnitude E), and is expressed as:

(1) $$v_{EO}=\mu_{EO}E=-\frac{\varepsilon_{m}\zeta_{W}}{\eta }E$$

(2) $$v_{EP,L}=\mu_{EP,L}E=\frac{\varepsilon_{m}\zeta_{P}}{\eta }E\;(\mathrm{weak}\;\mathrm{field}\;\mathrm{regime})$$

where v is the velocity, and $\mu_{EO}$ and $\mu_{EP,L}$ are the linear EO and EP mobilities, respectively. The terms $\varepsilon_{m}$ and η represent the permittivity and viscosity of the suspending medium, respectively; and $\zeta_{W}$ and $\zeta_{P}$ denote the zeta potential of the channel wall/liquid and the particle/liquid interfaces, respectively. The nonlinear EK phenomena considered in this study include dielectrophoresis (DEP) and EP_NL_, which exhibit the nonlinear dependence of their velocities with the magnitude of E. The expression for $v_{DEP}$ of a spherical particle is:

(3) $$v_{DEP}=\mu_{DEP}\nabla E_{rms}^{2}=\frac{r_{p}^{2}\varepsilon_{m}}{3\eta }Re\left[f_{CM}\right]\nabla E_{rms}^{2}$$

where $r_{p}$ denotes the particle radius, $Re\left[f_{CM}\right]$ is the real part of the Clausius–Mossotti factor, which accounts for the polarization effects, and $E_{rms}$ represents the root-mean-square value of the electric field magnitude.

The magnitude of the EP_NL_ velocity ($v_{EP,NL}$) is estimated after the assessment of the overall particle velocity ($v_{P}$), $v_{EO}$ and $v_{EP,L}$, using the following expression:

(4) $$v_{EP,NL}=v_{P}-v_{EP,L}-v_{EO}$$

with $v_{P}$ being generally measured experimentally with particle-tracking velocimetry experiments (PTV) in a DEP-free microchannel with a constant cross-section.

To classify the velocity dependence of EP_NL_ with the electric field magnitude, several analytical models utilize the dimensionless applied field strength coefficient (β) and the dimensionless Peclet (*Pe*) and Dukhin (*Du*) numbers. Relevant mathematical expressions for $v_{EP,NL}$ only exist for the two limiting cases of small *Pe* (Pe≪1) and high *Pe* (Pe≫1). For the intermediate cases, there are no articulated expressions. The expressions of $v_{EP,NL}$ for the two limiting cases are given below [51,52,53]:

(5) $$v_{EP,NL}^{\left(3\right)}=\mu_{EP,NL}^{\left(3\right)}E^{3}{\hat{a}}_{E}\;\mathrm{for}\;\beta \sim 1,\;\mathrm{arbitrary}\;Du,\;\mathrm{and}\;Pe\ll 1\;(\mathrm{moderate}\;\mathrm{field}\;\mathrm{regime})$$

(6) $$v_{EP,NL}^{\left(3/2\right)}=\mu_{EP,NL}^{\left(3/2\right)}E^{3/2}{\hat{a}}_{E}\;\mathrm{for}\;\beta >1,\;Du\ll 1\;\mathrm{and}\;Pe\gg 1\;(\mathrm{strong}\;\mathrm{field}\;\mathrm{regime})$$

where $\mu_{EP,NL}^{\left(n\right)}$ represents the mobility, and n indicates the dependence of $v_{EP,NL}$ with a magnitude of E, based on the operating conditions (see Table S1). In the present work, only the moderate field regime was considered ($E^{3}$ dependence), since this regime is the appropriate regime given the values of β, *Pe* and *Du* obtained under the employed experimental operating conditions (Table S1). 

**Figure 1:**
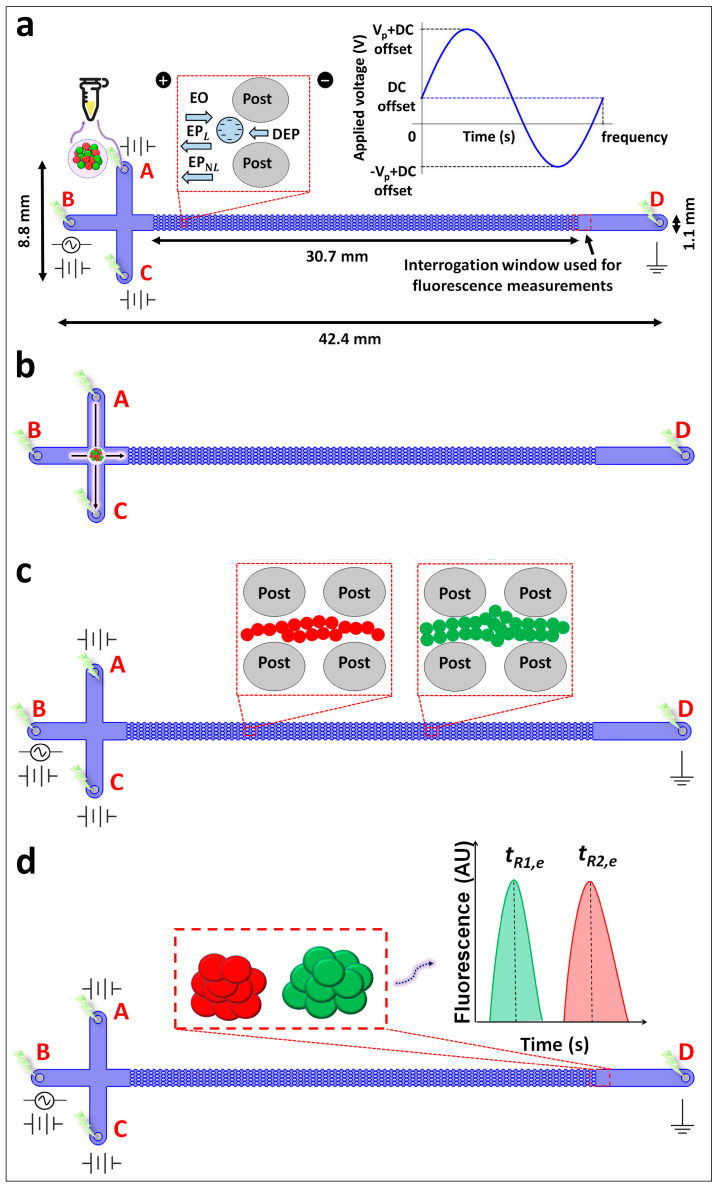
Schematic representation of the steps required for the cell separation process used in this study. (**a**) Illustration of the binary mixture of cells pipetted into the T-shaped iEK microchannel with four reservoirs labeled A–D. The channel dimensions and the location of the interrogation window used for the fluorescence measurements are indicated. The first figure inset depicts the four EK forces (EO, EP_L_, EP_NL_, and DEP) acting on the cells. The second figure inset contains a representation of the DC-biased AC voltage (500 (DC) + 600 ($V_{p}$) @ 0.4 Hz) employed in all the experiments. (**b**) Representation of the EK injection process, where a defined volume of the sample is electrokinetically injected into the main channel by application of electric voltages. (**c**) Illustration of the separation of the cells in the post array, where the formation of two “zones” of cell types is depicted, as the first cell type (green) is migrating faster than the second cell type (red). (**d**) Illustration of the elution of cells at the interrogation window, where the fluorescence of the samples is measured and analyzed to obtain the electropherograms for assessing the quality of the separations.

Thus, by considering all four EK phenomena, the overall particle velocity $(v_{P}$) in an iEK device, as represented in Figure 1a, becomes:

(7) $$v_{P}=v_{EO}+v_{EP,L}+{v_{DEP}+v}_{EP,NL}^{\left(3\right)}$$

The quality of each of the binary separations performed in this study was quantified by evaluating the electropherograms in terms of the separation resolution ($R_{s}$) which is expressed as:

(8) $$R_{s}=\frac{2(t_{R2,\;e}-t_{R1,e})}{W_{1}+W_{2}}$$

where W is the width of the peak at the base and $t_{R,e}$ is the experimental retention time of each cell type in the post array of the iEK device. The magnitudes of all four EK-phenomena depend on the properties of the channel, particle and suspending media, and on the local time-dependent electric field magnitude, as a DC-biased low-frequency AC signal was employed for all the separations.

## 3. Materials and Methods

### 3.1. Microdevices

All the devices were T-shaped iEK microchannels (Figure 1a) made from polydimethylsiloxane (PDMS, Dow Corning, Midland, MI, USA) using standard soft lithography techniques [43,54,55]. The PDMS casting of the microchannel was detached from the mold after curing, followed by punching holes for the inlet and outlet reservoirs. The device was then sealed with a PDMS-coated glass wafer by treating both with corona discharge. The depth of the iEK microchannel was 40 µm, and all the other channel dimensions are detailed in Figure 1a.

### 3.2. Suspending Medium and Cell Samples

A 0.2 mM solution of K_2_HPO_4_ was used as the suspending medium, Tween-20 was added at a low concentration of 0.05% (*v*/*v*) to avoid cytotoxic effects [56,57]. By adding 0.1 M KOH solution, the pH and conductivity of the suspending medium were adjusted to 7.1±0.6, and 43.1±2.8 μS/cm, respectively. These conditions yielded $\zeta_{W}$ of −60.1±3.7 mV, and $\mu_{EO}$ of (4.7±0.3)$\times 10^{-8}$ m^2^ V^−1^ s^−1^, respectively, which were characterized by current monitoring experiments [58]. Five types of cells (Table 1) possessing spherical and non-spherical shapes were selected based on characteristics with higher similarities compared to our prior work [50]. The cells studied here are *E. coli* (ATCC 11775), *B. subtilis* (ATCC 6051), *B. cereus* (ATCC 14579), *S. cerevisiae* (ATCC 9080), and *S. cerevisiae* (ATCC 9763). Standard methods were utilized for culturing and staining the cells using fluorescent SYTO dyes—Syto 85 (orange) nucleic acid stain and Syto 11 (green) nucleic acid stain (Thermo Fisher Scientific, Carlsbad, CA, USA) [43]. For all the experimentation, the exposure time of the cells to the suspending medium was limited to a short duration (<8 h) to avoid the development of cytotoxic effects [56,57]. The values of $\zeta_{P}$, $\mu_{EP,L}$ and $\mu_{EP,NL}^{\left(3\right)}$ for each cell type were independently experimentally assessed using PTV experiments (Table 1) in a channel with a constant cross-section (as described in Section S2 of Supplementary Materials) [34,59]. For all three separations, EK injection was used to introduce the binary mixture of cells into the iEK device [60].

### 3.3. Equipment and Software 

Four individual platinum wire electrodes (1.5 cm length and 0.584 mm diameter) labeled A−D (Figure 1) were employed to apply the electric potentials, which were programmed through a high-voltage power supply (Model HVS6000D, LabSmith, Livermore, CA, USA) using the LabSmith Sequencer software version 1.167. For all three distinct cell separations, the applied voltage sequence was the same, as described in Table 2. As reported in one of the prior studies by our group [61], under the conditions described in Table 2, the effects of the applied potential do not significantly affect the cell viability. The separation experiments were observed with a Zeiss Axiovert 40 CFL (Carl Zeiss Microscopy, Thornwood, NY, USA) inverted microscope and recorded as videos with a digital camera (Lumenera Infinity 2-1C camera model, Infinity Capture application software version 6.5.6) connected to the microscope.

### 3.4. Numerical Methods

The values of $\mu_{EP,L}$ and $\mu_{EP,NL}^{\left(3\right)}$ for each particle type (which are listed in Table 1 and Table S2) were numerically obtained by fitting the PTV data of the cell velocity as a function of the electric field to an analytical curve considering the cubic dependence of the EP_NL_ velocity on E (Figure S1). The fitting method was extended from our prior work [50] to include non-spherical cells (Table 1) and involved the use of a nonlinear regression and least squares method to estimate the mobilities. It is noteworthy that the values of $\mu_{EP,NL}^{\left(3\right)}$ are not a function of E under the evaluated operating conditions (Table S1), which is well in agreement with a recent study on the EP_NL_ of spherical colloidal particles [53]. A complete description of the method used to obtain these approximated values of $\mu_{EP,NL}^{\left(3\right)}$ is included in Section S2 of Supplementary Materials.

Numerical modeling of the stationary electric field within the device was performed using COMSOL Multiphysics 5.6 (COMSOL Inc., Burlington, MA, USA) based on the 2D device geometry depicted in Figure 1. The boundary and domain conditions used in the model are listed in Table S3 and Figure S2. From the complete stationary 2D solution, electric field magnitude data were collected for two conditions across the horizontal cutline (located at the center of the insulating posts array), as shown in Figure S3. For the first condition, the electric potential boundaries of reservoirs A and C were set to the values listed in Table 2 for the separation step only, while reservoirs B and D were set to ground. For the second condition, the electric potential boundaries of reservoirs A, C and D were set to ground, and a DC voltage of 100 V was modeled for reservoir B. The electric field data obtained across the cutline for both the conditions were used for training a brand-new regression algorithm to produce a continuous function of space and time for the electric field intensity and overall particle velocity, as well as a continuous function of time for the particle position. This enabled the prediction of the retention time ($t_{R,p}$) for each cell type under the voltage conditions listed in Table 2 for the separation step. A prediction of a range of retention times for each cell type, which takes into account the standard deviations of the experimentally measured post array dimensions and cell characteristics, was for the first time obtained by employing a continuous function for electric field intensity. The use of a continuous function significantly reduced the computational time and costs, with no loss of prediction accuracy. This is illustrated in Figure S4 by the comparison of the calculated values of E and $\left|\nabla E^{2}\right|$ across the cutline. The predicted retention times were then compared to the experimental retention time ($t_{R,\;e}$) for each cell type. To evaluate the overall particle velocity function (necessary to estimate the particle position and $t_{R,p}$), the previously determined characteristics of the cells listed in Table 1 were used. A fully detailed description of this algorithm is provided in Section S4.2 of Supplementary Materials.

The COMSOL model also allowed us to study the EK regime of the separation by assessing the independent impact of each EK phenomenon influencing the migration of the cells within the device. Shown in Figure S5 are plots of the individual particle velocities induced by each of the EK phenomena studied here.

### 3.5. Experimental Procedure

To ensure a stable EO flow, the microchannels were filled with the suspending medium before experimentation. For each separation, ~5 µL of the corresponding binary cell mixture (Table 3) was pipetted into reservoir A (Figure 1a), after which the four individual platinum wire electrodes were placed into the four reservoirs. All the separations involved a sequential application of three distinct sets of voltages (i.e., loading, gating, and injection; see Table 2) [60] to electrokinetically introduce the sample into the microchannel (Figure 1b–d). The fourth step of the EK injection process utilized a 500 V DC-biased 600 V peak amplitude at 0.4 Hz. Since the potential parameters of the frequency, peak amplitude and DC bias significantly affect the separation resolution [48], the DC-biased AC potential utilized in this study was chosen from a previous study where the potentials were fine-tuned to produce successful cell separations [50]. Therefore, the DC-biased AC potential (as indicated in Table 2) was applied for separating the cell mixtures with a higher degree of complexity compared to those utilized in our prior work. Each separation was considered complete when both cell types finished eluting as peaks from the post array. The electropherograms were built by plotting the fluorescence signal obtained from the elution of each cell type at the interrogation window (as shown in Figure 1d) with respect to the time. All the separations were repeated at least three times to confirm the reproducibility (Table S4, Figure S6).

## 4. Results and Discussion

### 4.1. Separation of Cells from Different Domains: E. coli and S. cerevisiae Cells 

**Figure 2:**
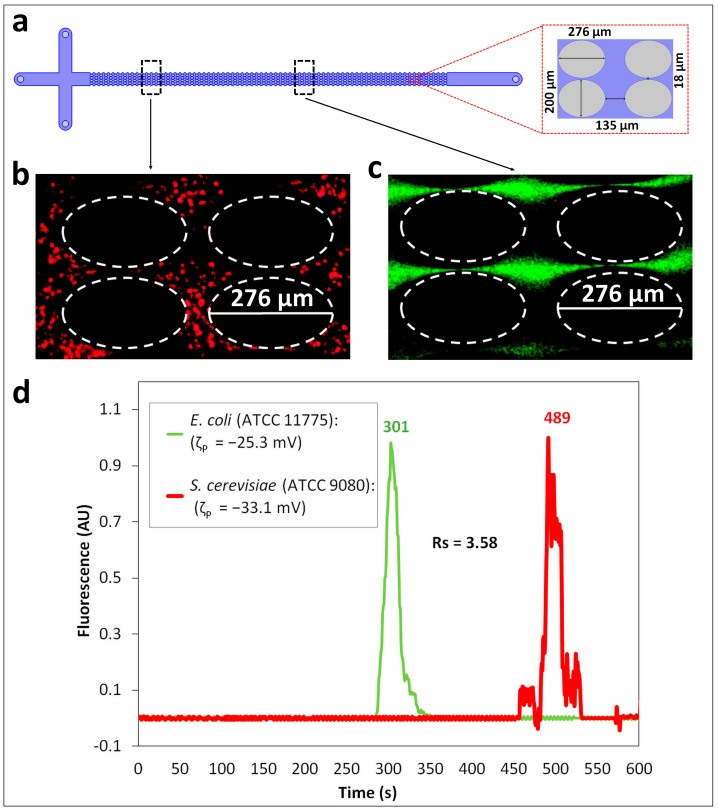
Separation ID 1 between *E. coli* (ATCC 11775) and *S. cerevisiae* (ATCC 9080) cells, which are from distinct domains, the prokaryotic and eukaryotic domains, respectively. (**a**) Schematic representation of the iEK device indicating the two different points of observation for the cells. (**b**,**c**) Images of the cells in the post array of the iEK device showing two different “zones” of cells as the *E. coli* (ATCC 11775, green) cells are migrating faster and ahead of the *S. cerevisiae* (ATCC 9080, red) cells (see Supporting Information Video S1). (**d**) Electropherogram of the separation built by analyzing the fluorescence signal at the end of the interrogation window. The applied DC-biased AC potential was V_p_ = 600 V, at 0.4 Hz and the DC bias was 500 V.

The first separation analyzed a binary mixture of prokaryotic *E.coli* (labeled green) and eukaryotic *S. cerevisiae* (ATCC 9080, labeled red) cells. The regression algorithm described in Section S4.2 of Supplementary Materials. Supplementary Data, utilizing the cell properties listed in Table 1 and the voltage conditions listed in Table 2, was used to predict the retention times for both cell types, as shown in Table 3. Based on previous work, a difference between the predicted retention times of at least 30 s (Δ*t_R_*_,*p*_ > 30 s) is required for a successful separation experiment [50]. The value of Δ*t_R_*_,*p*_, predicted by employing the voltage sequence listed in Table 2, was ~150 s, indicating that the separation would be possible. The experimental results of this separation are shown in Figure 2. The “zones” of *E. coli* (green) and *S. cerevisiae* (ATCC 9080, red) cells, which are formed while migrating across the post array at two different points of observation in the channel (Figure 2a), are shown in Figure 2b,c, where the *E. coli* cells were moving ahead of the *S. cerevisiae* cells. The two different points of observation (Figure 2a) for capturing the “zones” of the cells were chosen because the difference in the cell properties (Table 1) made the acquirement of the “zones” in the same window of observation highly challenging. The cell properties listed in Table 1 also explain the cell migration behavior, since under the low electric field conditions employed, the distinguishing EK phenomenon contributing to the differences in the overall cell velocities is electrophoresis. The *E. coli* cells, which possess lower magnitudes of $\zeta_{P}$ and $\mu_{EP,L}$, experience a lower magnitude of pull toward the inlet and hence migrate faster toward the outlet. All the cells in this study were negatively charged and thus, their electrophoretic migration was toward the inlet. In this case, the effects of EP_NL_, illustrated by the values of $\mu_{EP,NL}^{\left(3\right)}$, contributed to the separation, as these values follow the same trend as the $\zeta_{P}$ and $\mu_{EP,L}$ values, indicating a lower pull toward the inlet for the *E. coli* cells. This is supported by the electropherogram of this separation, shown in Figure 2d, where the *E. coli* cells eluted first, followed by the elution of the *S. cerevisiae* cells. These results, with a separation resolution of *R_s_* = 3.58, indicate a complete separation with well-resolved peaks. It is important to note that the peaks possess a non-Gaussian shape, which can be attributed to the use of DC-biased AC potentials, which cause the cells to move forward and backward within the microchannel. The experimental results had good reproducibility, with standard deviations below 4% between repetitions, as shown in Table S4 and the confidence interval plot of the electropherogram included in Figure S6a. Good agreement was obtained between the model-predicted and experimental retention time values, with deviations < 18% for both cell species (Table 3).

### 4.2. Separation of Cells from Same Domain and Different Species: B. subtilis and B. cereus Cells

**Figure 3:**
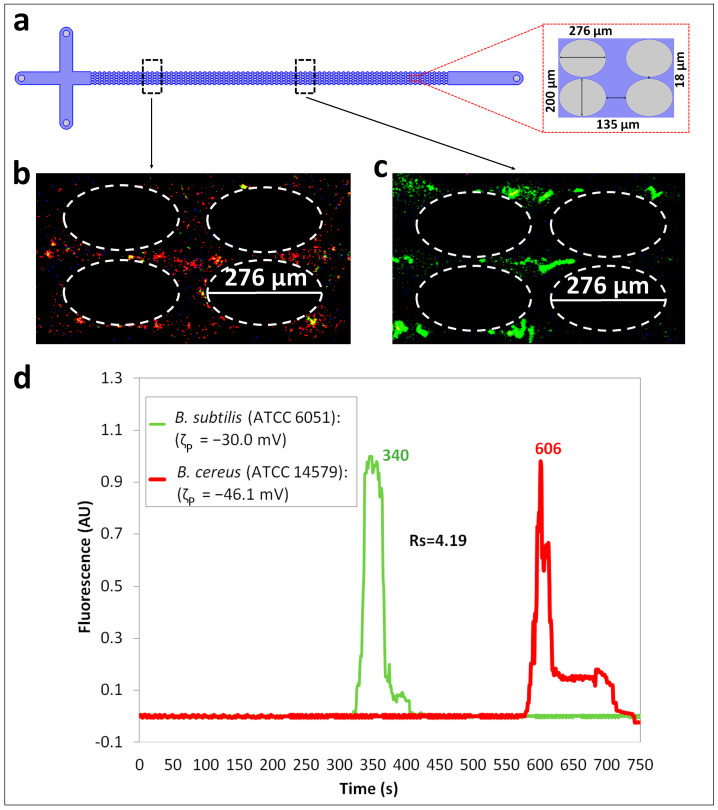
Separation ID 2 between *B. subtilis* (ATCC 6051) and *B. cereus* (ATCC 14579) cells, which are from the same prokaryotic domain but from distinct species. (**a**) Illustration of the iEK device indicating the two different points of observation for the cells. (**b**,**c**) Images of the migrating cells in the post array of the iEK device showing two “zones” where the *B. subtilis* (ATCC 6051, green) cells are ahead of the *B. cereus* (ATCC 14579, red) cells (see Supporting Information Video S2). (**d**) Electropherogram of the separation built by analyzing the fluorescence signal recorded at the end of the interrogation window. The applied DC-biased AC potential was V_p_ = 600 V, at 0.4 Hz and the DC bias was 500 V.

The second set of separation experiments investigated the discrimination between *B. subtilis* and *B. cereus* cells, which are cells from the same prokaryotic domain and distinct species. The *t_R_*_,*p*_ values for each cell type (Table 3) predicted through the regression algorithm, as described in Section S4.2 of Supplementary Materials, by the employing cell properties (Table 1) and voltage conditions (Table 2) indicated that the separation would be experimentally feasible as the Δ*t_R_*_,*p*_ was ~330 s. The experimental separation results at two different observation points in the post array (Figure 3a) are shown in Figure 3b,c. The characteristics of the *B. subtilis* and *B. cereus* cells and the overall particle velocity expression (Equation (7)) indicate that under low electric field conditions, electrophoresis contributes to the differences in the overall cell velocity, causing the *B. subtilis* cells to migrate faster than the *B. cereus* cells and thereby elute first from the insulating post array. Figure 3b,c show the cells as they migrate across the post array, where two distinct regions are seen: *B. subtilis* (green) cells are migrating ahead of the *B. cereus* (red) cells. Figure 3d shows the electropherogram of this separation, where the green peak denoting the elution of the *B. subtilis* species is seen first, followed by the red peak indicating the elution of the *B. cereus* cells. Thus, this confirms the expected results from the cell properties ($\zeta_{P}$ and $\mu_{EP,L}$) and the observations in Figure 3b,c. It is important to note that this separation would have had the opposite elution order under higher voltages, since the values of $\mu_{EP,NL}^{\left(3\right)}$ follow the opposite trend to the $\zeta_{P}$ and $\mu_{EP,L}$ values, that is, at higher voltages, *B. cereus* cells would have eluted first [62]. In this particular separation, the effects of the EP_NL_ are detrimental; however, since the difference in the $\zeta_{P}$ values is large (~16 mV), the detrimental effects of the EP_NL_ did not significantly affect the final outcome. This separation resulted in a high separation resolution of *R_s_* = 4.19, indicating a complete separation between the two cell types. Good reproducibility with deviations < 6% between the experimental repetitions was achieved, as shown in Table S4, and the confidence interval plot of the electropherogram is shown in Figure S6b. Good agreement between the predicted and experimental retention times was also obtained for both the cell types, with a maximum deviation of −7% (Table 3).

### 4.3. Separation of Cells from Same Domain, Same Species and Different Strains: Two Distinct Strains of S. cerevisiae Cells

**Figure 4:**
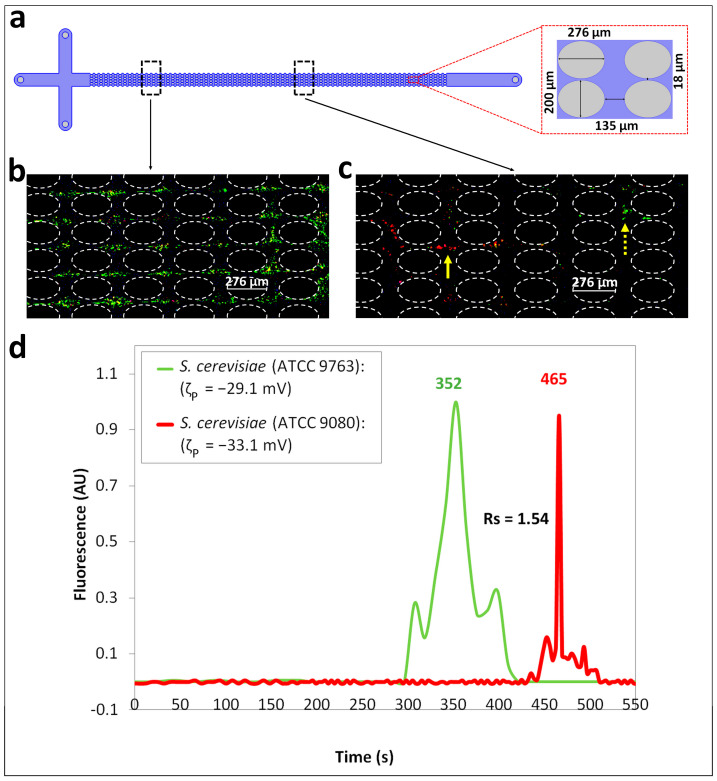
Separation ID 3 between *S. cerevisiae* (ATCC 9763) and *S. cerevisiae* (ATCC 9080) cells, which are closely related microbial strains from the same eukaryotic domain and same species. (**a**) Depiction of the iEK device indicating the two different points of observation for the cells. (**b**) Image of the cells appearing to be mixed, with no appreciable separation observed, at the first point of observation in the iEK device. (**c**) Image of the cells at the second point of observation in the post array of the iEK device, where the *S. cerevisiae* (ATCC 9080, green) cells are migrating faster than the *S. cerevisiae* (ATCC 9763, red) cells (see Supporting Information Video S3). (**d**) Electropherogram of the separation built by fluorescence signal analysis. The applied DC-biased AC potential was V_p_ = 600 V, at 0.4 Hz and the DC bias was 500 V.

The third and most difficult separation in this work was the discrimination between two closely related microbial strains of *S. cerevisiae* cells, which are cells from the same prokaryotic domain and same species. The experimental feasibility evaluated by the regression algorithm described in Section S4.2 of Supplementary Materials, employing the voltage conditions in Table 2, indicated that the separation would be experimentally feasible as the Δ*t_R_*_,*p*_ between the two yeast strains was ~100 s. Figure 4b,c show the experimental results of this separation observed at two different points across the iEK device (Figure 4a). Figure 4b shows that the cells were mixed, and no appreciable separation was taking place at the first point of observation in the iEK device. Figure 4c illustrates the formation of “zones” as the two cell species migrated across the posts array, since the properties of the cells (Table 1) used for this separation (Separation ID 3) are highly similar, capturing the formation of zones of cells within the same window of observation was possible. Based on the properties of the two *S. cerevisiae* cells, which are highly similar, the *S. cerevisiae* cells (ATCC 9763, labeled green) were expected to migrate toward the outlet faster than the *S. cerevisiae* cells (ATCC 9080, labeled red). This migration order was determined by the $\zeta_{P}$ and $\mu_{EP,L}$ of the cells, since the discriminating velocity components such as the $v_{EP,L}$ and $v_{EP,NL}$ of *S. cerevisiae* cells (ATCC 9763) and *S. cerevisiae* cells (ATCC 9080), respectively, favor the discrimination of cells under the employed conditions. In this case, the effects of the EP_NL_ contributed to the separation based on the values of the $\mu_{EP,NL}^{\left(3\right)}$, which follow the same trend as the $\zeta_{P}$ and $\mu_{EP,L}$ values, indicating a lower pull toward the inlet for the *S. cerevisiae* cells (ATCC 9763, labeled green). This migration behavior, where *S. cerevisiae* cells (ATCC 9763, labeled green) moved ahead, is indicated by a dotted yellow arrow in Figure 4c, followed by *S. cerevisiae* cells (ATCC 9080, labeled red), which is indicated by a solid yellow arrow in Figure 4c. The electropherogram in Figure 4d shows the green peak elution first, followed by the red peak. Since this separation involved almost identical cell types, as noted from their properties in Table 1, it was the most challenging in the present study. However, a separation resolution of *R_s_* = 1.54 was accomplished, indicating a complete separation between two closely related microbial strains of *S. cerevisiae* cells. The separation experiments, which were repeated three times to ensure the reproducibility, yielded good results, with <4% deviations between the experimental repetitions, as indicated in Table S4, and the confidence interval plot of the electropherogram is shown in Figure S6c. An agreement of <12.0% between the predicted and experimental retention times was obtained for both cell species (Table 3), highlighting that the model can be utilized as a valuable resource to design complex and highly challenging separations.

### 4.4. Insights from the Mathematical Model about the EK Mechanisms Driving Cell Separations

**Figure 5:**
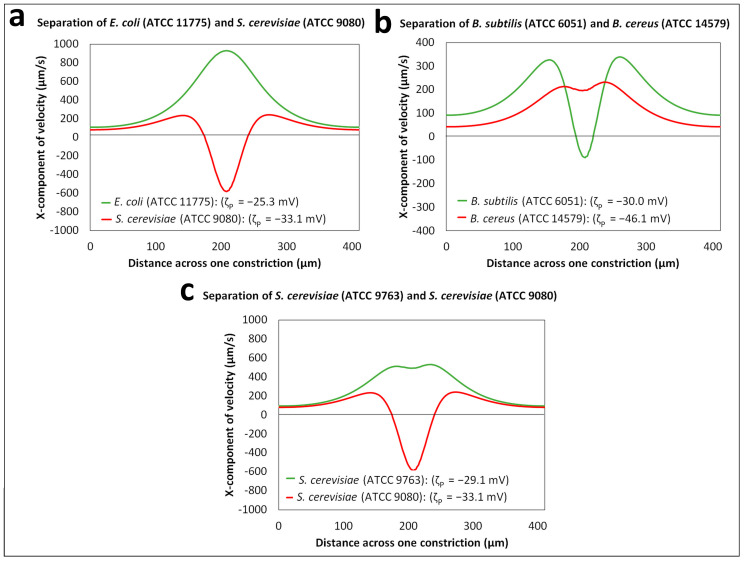
Prediction of the overall cell velocities at the peak amplitude (V(t = 0.625 s) = 1100 V) for a horizontal cutline (shown in Figure S3) across a constriction between two posts for all the separations: (**a**) separation ID 1: between *E. coli* (ATCC 11775) and *S. cerevisiae* (ATCC 9080) cells, (**b**) separation ID 2: between *B. subtilis* (ATCC 6051) and *B. cereus* (ATCC 14579) cells and (**c**) separation ID 3: between *S. cerevisiae* (ATCC 9763) and *S. cerevisiae* (ATCC 9080) cells.

The difference in the overall cell migration velocity (Equation (7)) is the main governing criterion for all the separations. The overall migration velocity of each cell type depends on the individual velocity components driven by the four EK phenomena present in the system, as expressed by Equations (1)–(6). For each separation set, the regression algorithm described in Section S4.2 of Supplementary Materials was used to predict the overall cell migration velocity for each cell type across a horizontal cutline between two posts (Figure S3). Since a DC-biased AC voltage (Table 2) was used for all the separations, the electric field distribution across the iEK device was time-dependent, and a maximum electric field magnitude was achieved at a time corresponding to the peak amplitude application. Figure 5 shows the overall cell migration velocity obtained at the maximum electric field magnitude for each of the two cell species considered in three distinct separation sets. The differences between the two overall cell migration velocities in each separation set illustrated the feasibility of the separation experiments and determined the discrimination between the two cell species under consideration.

The numerical COMSOL model was also utilized to assess the EK regime under which the separations took place. The estimations of the velocity components across a horizontal cutline between two posts (Figure S3) showed the effect of each of the four EK phenomena (Figure S5). As seen, all the separations were governed by differences in the cell velocities, where the electrophoresis contribution occurred in such a way that the discrimination between the cell types was enhanced, confirming the mechanisms behind the observed elution order of the cells. It is important to note that under the selected operating conditions (Table 2), the EO phenomena, the most dominant phenomena in most regions of the iEK device, is not a discriminatory mechanism. The EO velocity was the same for all the cells, the magnitude of the DEP velocity was minimal for all the cells, and the differentiating EK phenomena was mainly electrophoresis (EP_L_ and EP_NL_). For the specific case of the Separation ID 2, the effects of the EP_NL_ worked against the separation. However, by combining the effects of the EP_L_ and EP_NL_, the final outcome of Separation ID 2 was not significantly affected. It is important to highlight that the combination of the EP_L_ and EP_NL_ effects, a unique ability of iEK systems, was crucial in achieving all the separations. If only linear EK (EO and EP_L_) effects were considered, as seen from the cell properties (Table 1), each separation set would have been extremely difficult or perhaps not feasible. Thus, the initiation of nonlinear EK (especially EP_NL_) effects in the regions around the insulating posts, which have higher electric field intensities than those regions away from the posts, has enabled designing and performing sophisticated separation schemes for highly challenging sample mixtures. Given these valuable insights about the EK mechanisms of the cell separations and the fact that no empirical correction factors are needed to match the predicted and experimental results [63], the mathematical model hence serves as a useful resource for designing effective separation strategies. Potential causes of the observed deviations between the modeled and experimental results include local electric field distortions caused by cells, cell interactions, and EK injection bias during sample injection [60,64], since these effects are currently not included in the model. These results leveraged the findings from two previous reports on fine-tuning the characteristics of the applied AC potential [48] and iEK device [49]. The findings of this study extended the limits of DC-biased AC-iEK systems to separate spherical and non-spherical cell mixtures with complexities ranging from cells from different domains to cells from the same species.

## 5. Conclusions

Presented here are three distinct separations of binary mixtures of cells, with an increasing order of difficulty, in an iEK microchannel stimulated by a low-frequency DC-biased AC voltage. This study is the first demonstration of the application of DC-biased AC-iEK systems for successfully separating three mixtures of spherical and non-spherical yeast and bacterial cells, performed with a higher degree of complexity, including the separation of cells of the same species, within a matter of few minutes. Mathematical modeling with COMSOL Multiphysics and data regression analysis using analytical curve fitting of the cell velocity with the electric field magnitude and a continuous function for the electric field intensity were utilized to improve the computational time for predicting the retention time of each cell type. The model also guided experimentation by assessing the experimental feasibility of the three distinct separations under the selected DC-biased AC voltage. The model also provided valuable insights about the effect of the four EK phenomena on the overall cell migration behavior. The good agreement (deviations < 18%) between the predicted and experimental results for all the separations highlighted that the model can be utilized for designing effective separation strategies. Each of the three separations achieved separation resolution *R_s_* > 1.5, indicating complete separation between the two cell species under consideration. Good reproducibility, with deviations < 6% between experimental repetitions, was achieved for all the cases. This is the first report demonstrating the separation of closely related microbial strains by applying a low-frequency DC-biased AC voltage in an iEK device while considering the EP_NL_ effects. Thus, this investigation emphasizes the potential of iEK systems to design and perform challenging microbial separations, along with the capability of these systems to keep cells viable post separation. This study unravels further research opportunities related to iEK systems for separating microorganisms by employing DC-biased AC potentials and illustrates the need to further study the effects of the frequency, peak amplitude and DC bias on the cell viability and separation resolution. Future extensions of this study will include evaluating the effect of the microchannel wall on the resolution of separations and extending the applications of DC-biased AC-iEK systems to complex biological mixtures containing different suspending media and involving three or more cell types. Furthermore, quantitative cell analysis will also be explored to determine the capacity of iEK systems for the enrichment of cells and target analytes. 

## Figures and Tables

**Table 1:** Characteristics of the cells used in this study.

Cell ID	Dimensions (μm)	$\zeta_{P}$ (mV)	$\mu_{EP,L}$ × 10^−8^ (m^2^V^−1^s^−1^)	$\mu_{EP,NL}^{\left(3\right)}$ × 10^−18^ (m^4^V^−3^s^−1^)
*E. coli* (ATCC 11775)	3.2 ± 0.3 long 1.1 ± 0.2 wide	−25.3 ± 2.1 ^1^	−1.97 ± 0.1 ^1^	−2.1 ± 0.1 ^1,2^
*B. subtilis* (ATCC 6051)	7.7 ± 1.1 long 1.8 ± 0.3 wide	−30.0 ± 5.8 ^1^	−2.34 ± 0.4 ^1^	−17.2 ± 1.9 ^1,2^
*B. cereus* (ATCC 14579)	4.8 ± 0.5 long 1.5 ± 0.2 wide	−46.1 ± 3.1 ^1^	−3.50 ± 0.2 ^1^	−3.9 ± 0.1 ^1,2^
*S. cerevisiae* (ATCC 9080)	5.8 ± 0.5 diameter	−33.1 ± 4.8 ^1^	−2.58 ± 0.4 ^1^	−24.1 ± 4.1 ^1,2^
*S. cerevisiae* (ATCC 9763)	7.0 ± 0.7 diameter	−29.1 ± 3.7 ^1^	−2.26 ± 0.3 ^1^	−9.0 ± 0.1 ^1,2^

^1^ The values of $\zeta_{P}$ $\mu_{EP,L}$, and $\mu_{EP,NL}^{\left(3\right)}$ were specific to the suspending medium employed in this work. ^2^ The values of $\mu_{EP,NL}^{\left(3\right)}$ were approximated by fitting an analytical curve of the cubic dependence of EP_NL_ velocity on E (for more details on the approximation, please see Figure S1 and Table S2).

**Table 2:** Voltage conditions used for the EK sample injection and all the DC-biased AC-iEK-based cell separations.

Step	Run Time (s)	Applied Voltage (V) in Each Reservoir			
		A	B	C	D
Loading (DC)	10	500	300	0	500
Gating (DC)	5	1000	1000	1000	0
Injection (DC)	5	200	500	200	0
Separation (AC + DC bias)	700	200	$500\;(\mathrm{DC})+600\;(V_{p}$) @ 0.4 Hz	200	0

**Table 3:** Results of all the cell separations performed in this study: separation resolution ($R_{s}$), predicted retention time ($t_{R,p}$) compared with the experimental retention time ($t_{R,e}$) and deviation between $t_{R,p}$ and $t_{R,e}$ for all the cell separations.

Separation ID and Description	Cell IDs	*R_s_*	Predicted *t_R_*_,*p*_ (s)	Experimental *t_R_*_,*e*_ (s)	Deviation of *t_R_*_,*p*_ vs. *t_R_*_,*e*_ (%)
1 Separation of cells from different domains	*E. coli* (ATCC 11775)	3.58	252.1 ± 4.1 ^1^	297.0 ± 5.0	15.1 ± 1.3 ^1^
	*S. cerevisiae* (ATCC 9080)		401.8 ± 4.6 ^1^	489.3 ± 15.9	17.8 ± 0.9 ^1^
2 Separation of cells from same domain and different species	*B. subtilis* (ATCC 6051)	4.19	343.9 ± 7.6 ^1^	330.3 ± 9.5	−4.1 ± 0.3 ^1^
	*B. cereus* (ATCC 14579)		674.4 ± 14.9 ^1^	634.0 ± 32.7	−6.4 ± 2.3 ^1^
3 Separation of cells from same domain, same species and different strains	*S. cerevisiae* (ATCC 9763)	1.54	301.6 ± 6.5 ^1^	342.7 ± 10.5	12.0 ± 1.8 ^1^
	*S. cerevisiae* (ATCC 9080)		401.8 ± 4.6 ^1^	456.0 ± 7.3	11.9 ± 1.0 ^1^

^1^ The predicted values have a range as the standard deviations of the experimentally measured $\zeta_{P}$, $\mu_{EP,NL}^{\left(3\right)}$ and post size were included in the predictions.

## Data Availability

The data presented in this study are available on request from the corresponding author.

## Supplementary Materials

The following supporting information can be downloaded at https://www.mdpi.com/article/10.3390/bios14050237/s1; Table S1: Values of the parameters used to analyze the moderate field regime, cubic dependence ($E^{3}$); Table S2: Electrokinetic mobilities obtained with direct curve fitting of theoretical cubic dependence of cell velocity with electric field magnitude and fitting metrics; Table S3: Information about the domain and boundary conditions defined in the model. Domains are depicted in Figure S2 and the labels A, B, C, and D, are used to indicate the electrodes. Details on the voltages used for the EK injection and separation process are reported in Table 2 of the manuscript; Table S4: Values of the retention times for three distinct experimental repetitions of each separation; Figure S1: Curve fitting of the experimental velocity profile for the cell species shown in Table S2 as a function of the electric field magnitude in the SY cubic model; Figure S2: Representation of the domains and boundaries used in the computational model; Figure S3: Illustration of the horizontal cutline utilized to predict the electric field and velocity data for all the cell types investigated in this study; Figure S4: Results of curve fitting using a Fourier series expansion of the E-field profile; Figure S5: Predicted overall and individual cell velocities exerted by the four EK phenomena across the cutline (Figure S3) for all the cell separations (separation IDs 1–3); Figure S6: Confidence interval plots indicating the reproducibility between experiments for all the cell separations (separation IDs 1–3); Video S1: Separation of cells from different domains; Video S2: Separation of cells from same domain and different species; Video S3: Separation of cells from same domain, same species and different strains. References [65,66] are cited in the supplementary materials.
