# Supplementary material for: Development of a DC-Biased AC-Stimulated Microfluidic Device for the Electrokinetic Separation of Bacterial and Yeast Cells
Source: Biosensors (Basel). 2024 May 9;14(5):237. doi: 10.3390/bios14050237 (PMC11117482; doi:10.3390/bios14050237)
Supplement: Supplementary file 1 [file biosensors-14-00237-s001.zip › Supplementary material_R1_01.pdf]

Supplementary Material

Development of a DC-biased AC stimulated microfluidic device for the electrokinetic separation of bacterial and yeast cells

Nuzhet Nihaar Nasir Ahamed[a], Carlos A. Mendiola-Escobedo[b], Victor H. Perez-Gonzalez[b,*] and Blanca H. Lapizco-Encinas[a,*]

[a] Microscale Bioseparations Laboratory and Biomedical Engineering Department, Rochester Institute of Technology, 160 Lomb Memorial Drive, Rochester, New York, 14623, United States.

[b] School of Engineering and Sciences, Tecnologico de Monterrey, Monterrey, Nuevo Leon 64849, Mexico.

Correspondence should be addressed to the following authors:
Victor H. Perez-Gonzalez (PhD)
Email: vhpg@tec.mx

Blanca H. Lapizco-Encinas (PhD)
Email: bhlbme@rit.edu

1. Contents

1. EVALUATION OF THE ELECTRIC FIELD REGIME FOR NONLINEAR ELECTROPHORETIC VELOCITY: INCLUDES TABLE S1 .. 2

2. ANALYTICAL CURVE FITTING OF THE CUBIC DEPENDENCE OF EP_{NL} VELOCITY ON E: INCLUDES TABLE S2 AND FIGURE S1 2

3. INFORMATION ON THE COMPUTATIONAL MODEL: INCLUDES FIGURE S2, AND TABLE S3 6

4. ESTIMATION OF THE PREDICTED RETENTION TIME USING THE COMPUTATIONAL MODEL: INCLUDES FIGURES S3-S4. 7

4.1 REPRESENTATION OF THE HORIZONTAL CUTLINE USED FOR VELOCITY ESTIMATION: 7

4.2 INFORMATION ON THE REGRESSION ALGORITHM USED FOR $T_{R,P}$ PREDICTION: 7

5. COMPARISON OF THE VELOCITY MAGNITUDES OF ALL FOUR EK PHENOMENA: INCLUDES FIGURE S5. 10

6. REPRODUCIBILITY BETWEEN REPETITIONS: INCLUDES TABLE S4 AND FIGURE S6 11

7. REFERENCES 12

1. Evaluation of the electric field regime for nonlinear electrophoretic velocity: includes Table S1

To classify the velocity dependence of nonlinear electrophoresis (EP_{NL}) with electric field magnitude, and to identify the appropriate field regime of the nonlinear electrophoretic velocity, the three dimensionless parameters (β, Du and Pe) utilized are expressed as follows [1–3] :

$$\beta = \frac{Ea}{\varphi} \quad \text{(S1)}$$

$$Du = \frac{K^{\sigma}}{K_m a} \quad \text{(S2)}$$

where E denotes the applied electric field magnitude, a is the particle radius, φ is the thermal voltage, $K^{\sigma}$is surface conductivity, K_m is bulk conductivity, $v_{EP,L}$ and $v_{EP,NL}$ represent the linear and nonlinear electrophoretic particle velocity, respectively, and D is the diffusion coefficient.

Table S1. Values of the parameters used to analyze the moderate field regime, cubic dependence (E^3).

Separation ID	Cell ID – Label color	β	Du	Pe	E used for parameters estimation (V/cm)	Dependence of nonlinear EP with E
1	*E. coli* (ATCC 11775) - Green	0.3	0.09	0.1	100	Moderate (E^3)
	S. cerevisiae (ATCC 9080) - Red	0.9	0.03	0.4	50	Moderate (E^3)
2	*B. subtilis* (ATCC 6051) - Green	0.7	0.05	0.3	100	Moderate (E^3)
	B. cereus (ATCC 14579) - Red	0.5	0.10	0.3	100	Moderate (E^3)
3	*S. cerevisiae* (ATCC 9763) - Green	0.8	0.02	0.3	50	Moderate (E^3)
	S. cerevisiae (ATCC 9080) - Red	0.9	0.03	0.4	50	Moderate (E^3)

2. Analytical curve fitting of the cubic dependence of EP_{NL} velocity on E: includes Table S2 and Figure S1

Under the SY model [2,3], a dielectric particle, whose zeta potential (negative) is lower in absolute value than the absolute value of the zeta potential of the channel wall (negative), will travel in the direction of an applied electric field ($\mathbf{E}$) when suspended in an electrolyte solution. In such a case, the magnitude of the particle velocity presents two types of dependencies with the electric field. One dependency is based on electric field magnitude (E), related to electroosmotic (EO) flow and electrophoresis (EP), and the other dependency is linked to $|\nabla E^2|$, related to dielectrophoresis (DEP). For the case of EP, corrections over the classic linear dependence lead to a nonlinear response whose power depends on the particle-solution interface properties [2,3]. Each response can be associated to a mobility (μ), such that:

$$v_p = \left(\mu_{EO} + \mu_{EP,L}\right)E + \mu_{EP,NL}^{(3)}E^3 + \mu_{DEP}|\nabla E^2| \quad \text{(S3)}$$

where the nonlinear term was proven to be cubic in the previous section for every cell species analyzed in this

work.

To obtain the value of the mobility associated with nonlinear electrophoretic (EP) velocity, particle tracking velocimetry (PTV) experiments were performed over channels with no insulating structures (flat channels) subjected to DC electric fields ranging from 25-1200 V/cm, enabling the construction of their speed profile as function of E (as shown in **Figure S1**). Such channels generate in principle a constant electric field profile, resulting in $|\nabla E^2| = 0$, and in the annulation of the DEP term in eqn. (S3).

Now, since the nonlinear electrophoretic response is obtained when expanding particle speed in powers of E or in powers of Du, it is possible to use the associated coefficients of these corrections as fitting parameters to experimental data [2,3]. In this way, the theoretical particle velocity profile can be obtained using a regression taking only linear and cubic terms. Let v_i be a vector that contains the information of the particle velocity associated with the electric field magnitude domain E_i; E_{ij} the design matrix in the electric field domain; μ_j the parameter vector that contains the information of the mobilities; and ε_i an error vector; then:

$$v_i = E_{ij}\mu_j + \varepsilon_i \tag{S4}$$

Let p be the total number of points in the experimental velocity profile. For the present problem, $(\mu_{EO} + \mu_{EP,L})$ and $\mu_{EP,NL}^{(3)}$ are to be used as fitting parameters such that eqn. (S4) can be rewritten as:

$$\begin{bmatrix} v_1 \\ \vdots \\ v_p \end{bmatrix} = \begin{bmatrix} E_1 & E_1^3 \\ \vdots & \vdots \\ E_p & E_p^3 \end{bmatrix} \begin{bmatrix} (\mu_{EO} + \mu_{EP,L}) \\ \mu_{EP,NL}^{(3)} \end{bmatrix} + \begin{bmatrix} \varepsilon_1 \\ \vdots \\ \varepsilon_p \end{bmatrix} \tag{S5}$$

Then, with the use of the least-squares estimation, the predicted value of the mobilities ($\hat{\mu}_j$) can be obtained with:

$$\hat{\mu}_j = \left(E_{ij}^{\mathrm{T}} E_{ij}\right)^{-1} E_{ij}^{\mathrm{T}} v_i \tag{S6}$$

and,

$$\hat{v}_i = E_{ij}\hat{\mu}_j \tag{S7}$$

with $\hat{v}_i$ being the predicted value of particle speed [5].

The deviation of such estimation is obtained by means of the variance-covariance matrix of the parameters in $\hat{\mu}_j$, whose definition is:

$$Var(\hat{\mu}_j) = \sigma^2 \left(E_{ij}^{\mathrm{T}} E_{ij}\right)^{-1} = \left(v_i - E_{ij}\hat{\mu}_j\right)^{\mathrm{T}} \left(v_i - E_{ij}\hat{\mu}_j\right) \frac{\left(E_{ij}^{\mathrm{T}} E_{ij}\right)^{-1}}{i-2} \tag{S8}$$

The 11 term in such matrix provides the variance of the mobility associated with the linear response, and the jj term provides the variance of the mobility associated with the nonlinear response [5].

The metric used to determine the accuracy of the regression model and its capability to make predictions within the electric field domain is the coefficient of determination R^2, defined as:

$$R^2 = 1 - \frac{\sum_{i=1}^{p} \left(v_i - E_{ij}\hat{\mu}_j\right)^2}{\sum_{i=1}^{p} (v_i - \bar{v})^2} \tag{S9}$$

in which $\bar{v}$ is the mean value of the particle velocity profile [5].

This curve fitting method is also used to determine the mobility associated with linear EP only differing in the amount of fitting parameters and in the E domain taken to perform it. Now, since μ_{EO} is coupled in the linear response, current monitoring experiments were also done in flat channels resulting in $\mu_{EO} \approx 4.7 \times 10^{-8}\ \mathrm{m^2 V^{-1} s^{-1}}$. With this, it is possible to solve for $\mu_{EP,L}$ when the curve fitting is complete.

This method was applied to five cell types used in this study. The estimation of their mobilities of linear and nonlinear EP, their respective standard deviations and coefficients of determination are contained in **Table S2.** Their curve fittings are illustrated in **Figure S1**. It is important to note that the values of $\mu_{EP,NL}^{(3)}$ are constant and not a function of electric field under the evaluated operating conditions (**Table S1**),which are in agreement with

the trends provided by a recent study by Cobos et. al on EP_{NL} phenomena for a spherical colloidal particle [1].

Table S2. Electrokinetic mobilities obtained with direct curve fitting of theoretical cubic dependence of cell velocity with electric field magnitude and fitting metrics.

Cell ID	$\mu_{EP,L} \times 10^{-8}$ ($m^2V^{-1}s^{-1}$)	$\mu^{(3)}_{EP,NL} \times 10^{-17}$ ($m^4V^{-3}s^{-1}$)	R^2
E. coli (ATCC 11775)	-1.97 ± 0.10	-0.21 ± 0.01	0.899
S. cerevisiae (ATCC 9763)	-2.26 ± 0.30	-0.76 ± 0.17	0.983
B. subtilis(ATCC 6051)	-2.34 ± 0.46	-1.72 ± 0.19	0.965
B. cereus (ATCC 14579)	-3.59 ± 0.24	-0.39 ± 0.01	0.965
S. cerevisiae (ATCC 9080)	-2.58 ± 0.37	-2.41 ± 0.41	0.911

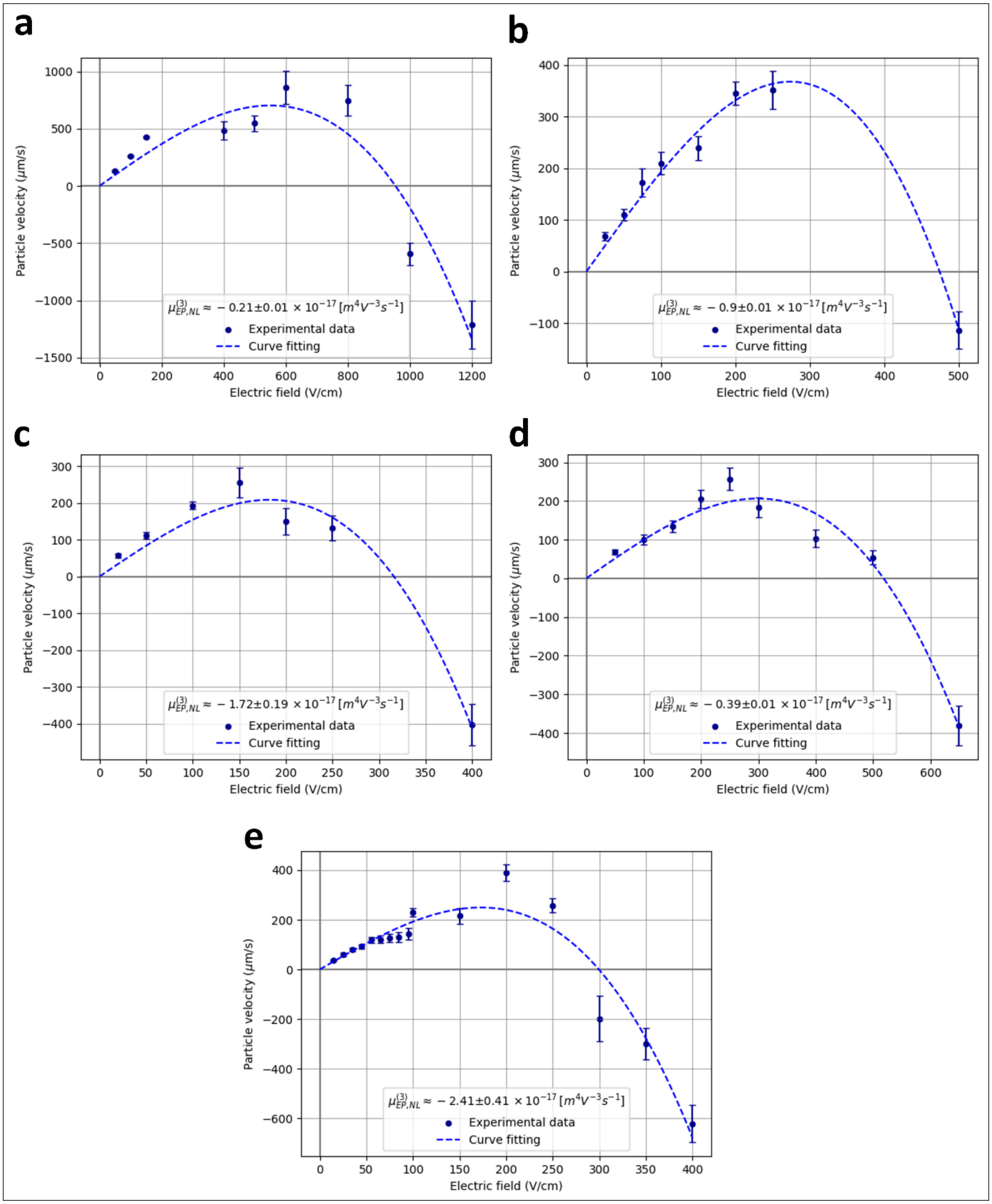

Figure S1. Curve fitting of the experimental velocity profile for the cell species shown in **Table S2** as a function of the electric field magnitude in the SY cubic model. The analyses are illustrated for **(A)** *E. coli* (ATCC 11775), **(B)** *S. cerevisiae* (ATCC 9763), **(C)** *B. subtilis* (ATCC 6051), **(D)** *B. cereus* (ATCC 14579), and **(E)** *S. cerevisiae* (ATCC 9080).

3. Information on the computational model: includes Figure S2, and Table S3

A 2D computational model built using COMSOL Multiphysics was considered to be an appropriate choice for the modeling [4], mainly because the changes in the electric field distribution along the channel depth can be ignored. The Electric Currents module within COMSOL Multiphysics was utilized for estimating the electric field distribution across the iEK device and for solving the Laplace equation. The relative permittivity and conductivity of the fluid suspending medium (ε_r) were set to 78.4 and 4.07 x 10^{-3} S/m (40.7 µS/cm), respectively. **Figure S2** depicts the different boundary conditions and domains used, and **Table S3** lists these conditions as equations. Maximum and minimum element sizes for free triangular meshes were 130 µm and 0.261 µm, respectively.

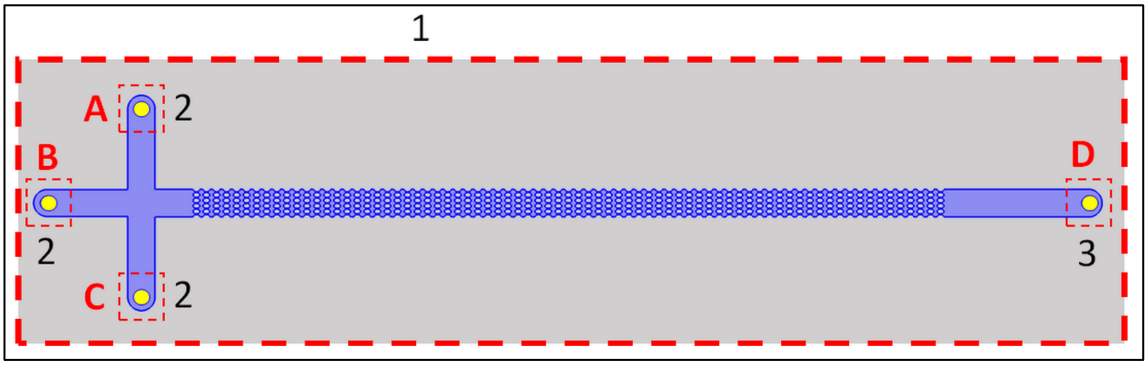

Figure S2. Representation of the domains and boundaries used in the computational model. Each colored area shows each of the different domains employed and the red dotted boxes indicate the boundaries used. The gray color illustrates the PDMS domain, the blue color represents the suspending medium field, and the yellow color indicates the platinum electrodes. The labels A, B, C, and D indicate electrodes used in the EK injection and separation process. The numbers 1, 2, and 3 represent the boundaries listed in **Table S3**. Boundary 1 represents the outer PDMS surface; boundary 2 describes the three reservoirs where electric potentials are applied: A, B, and C. Boundary 3 is the ground electrode D. All the other channel dimensions are shown in **Figure 1** in the manuscript.

Table S3. Information about the Domain and Boundary conditions defined in the model. Domains are depicted in **Figure S2** and the labels A, B, C, and D, are used to indicate the electrodes. Details on the voltages used for the EK injection and separation process are reported in **Table 2** of the manuscript.

Domain conditions		
**Domain type**	**Element region & color**	**Definition***
Current conservation	PDMS domain (channel walls and insulating posts), gray color	$\nabla \cdot \mathbf{J} = -\nabla \cdot \left(\left(\sigma + \varepsilon_0 \varepsilon_r \frac{\partial}{\partial t} \right) \mathbf{E} \right)$; where $\mathbf{E} = -\nabla V$
	Suspending medium domain, blue color	
	Platinum electrodes domain, yellow color	
Initial values	PDMS domain (channel walls and insulating posts), gray color	$V_0 = 0$
	Suspending medium domain, blue color	
	Platinum electrodes domain, yellow color	

Boundary conditions		
**Boundary condition type**	**Element number**	**Definition***
Electric insulation	1	$\mathbf{n} \cdot \mathbf{J} = 0$
Electric potential	2	$V_A = V_{applied,A}$; $V_B(t) = V_{DC} + V_p \sin(\omega t)$; $V_C = V_{applied,C}$; $V_D = V_{applied,D}$;

*V represents the electric potential, featuring a temporal component, V_p is the peak amplitude of the applied electric potential in the DC-biased (V_{DC}) AC signal, **J** is the electric current density and $\omega = 2\pi f$, with f representing the AC frequency. The variables ε_0 and ε_r represent the permittivity of the vacuum and the relative permittivity of each given domain, respectively. The permittivity of a given domain is $\varepsilon = \varepsilon_0 \varepsilon_r$.

4. Estimation of the predicted retention time using the computational model: includes Figures S3-S4.

4.1 Representation of the horizontal cutline used for velocity estimation:

A horizontal cutline, 411 μm long, spanning across a constriction between two posts is set in the middle of the post array, as shown in **Figure S3**. Based on the electric field and velocity estimation obtained across the cutline, the total retention time ($t_{R,p}$) for each cell type to migrate across the cutline was predicted. As this data is provided by the selected mesh in COMSOL, it should be appropriate to fit an analytical curve by considering the regions between points to attain a better prediction. After determining this information for one constriction, the same process is repeated until the position of the cell type is greater than or equal to the overall length of the array. This lays the foundation for the algorithm used to predict $t_{R,p}$.

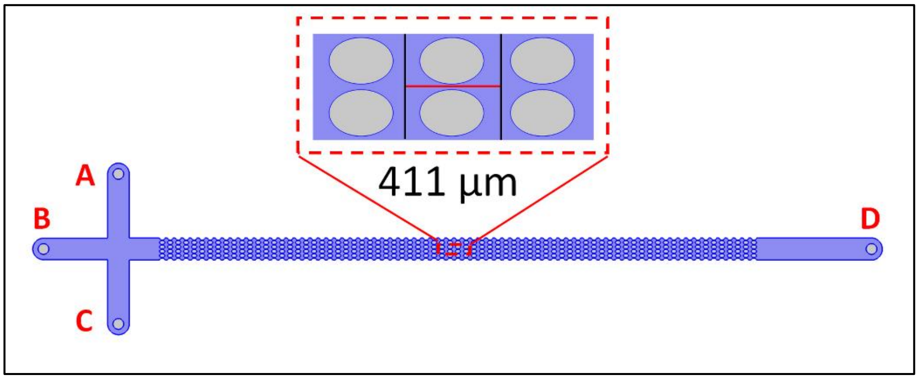

Figure S3. Illustration of the horizontal cutline utilized to predict electric field and velocity data for all the cell types investigated in this study. The length of the cutline considered is 411 µm, as the posts are 276 µm wide, and each lateral constriction is 135 µm, the total cutline length is 67.5 µm + 276 µm + 67.5 µm = 411 µm.

4.2 Information on the regression algorithm used for $t_{R,p}$ prediction:

Due to the presence of insulating structures within the channel, the E and $|\nabla E^2|$ profiles get amplified, thereby generating functions of the position in the defined cutline (x). To predict particle position (determine $\hat{r}_p$) along the microchannel illustrated in **Figure S3**, it is necessary to find analytic curves to both E and $|\nabla E^2|$ due to the direct dependency of particle velocity in these quantities as described in Equation (S3). The use of analytic curves is crucial to obtain the best approximation to the arising differential equation:

$$\frac{d}{dt}\hat{r}_p(t) = \hat{v}_p(x,t) \qquad \text{(S10)}$$

and because particle velocity is a function of both position and time. The profiles have two properties that must be satisfied in the analytical curves: (a) at least within the post section of the channel, they are periodic and (b) the derivative with respect to x is continuous in the boundaries between constrictions. Properties that are naturally satisfied by their Fourier series expansion, with the period being defined as the length of one constriction (L). Thereby, the predicted electric field ($\hat{E}$) can be defined as:

$$\hat{E}(x) = a_0 + \sum_{n=1}^{N}\left[a_n \sin\left(\frac{2n\pi}{L}x\right) + b_n \cos\left(\frac{2n\pi}{L}x\right)\right] \qquad \text{(S11)}$$

in which:

$$a_0 = \frac{1}{L}\int_0^L E(x)\, dx \quad \text{(S12)}$$

$$a_n = \frac{2}{L}\int_0^L E(x)\sin\left(\frac{2n\pi}{L}x\right)\, dx \quad \text{(S13)}$$

$$b_n = \frac{2}{L}\int_0^L E(x)\cos\left(\frac{2n\pi}{L}x\right)\, dx \quad \text{(S14)}$$

However, the predicted $|\nabla E^2|$ profile ($\hat{G}^{(2)}$) will be obtained by differentiation of the $E^2(x)$ series expansion, to use a single set of points and not lose any information by performing a numerical derivative. Since the problem is reduced to the x-axis, the gradient becomes one dimensional, and:

$$\hat{G}^{(2)}(x) = |\widehat{\nabla E^2}| = \frac{d}{dx}\left\{A_0 + \sum_{n=1}^{N}\left[A_n \sin\left(\frac{2n\pi}{L}x\right) + B_n \cos\left(\frac{2n\pi}{L}x\right)\right]\right\} \quad \text{(S15)}$$

where the superscript (2) references that this quantity is related to the gradient of the electric field squared and:

$$A_0 = \frac{1}{L}\int_0^L E^2(x)\, dx \quad \text{(S16)}$$

$$A_n = \frac{2}{L}\int_0^L E^2(x)\sin\left(\frac{2n\pi}{L}x\right)\, dx \quad \text{(S17)}$$

$$B_n = \frac{2}{L}\int_0^L E^2(x)\cos\left(\frac{2n\pi}{L}x\right)\, dx \quad \text{(S18)}$$

When the derivative in eqn. (S15) is performed, $\hat{G}^{(2)}(x)$ becomes:

$$\hat{G}^{(2)}(x) = \frac{2n\pi}{L}\sum_{n=1}^{N}\left[A_n \cos\left(\frac{2n\pi}{L}x\right) - B_n \sin\left(\frac{2n\pi}{L}x\right)\right] \quad \text{(S19)}$$

which uses the same coefficients of the Fourier expansion of $E^2(x)$ and the use of a single set of points to carry out both curve fittings.

Using the same procedure to obtain eqn. (S13), the $\hat{G}^{(1)}$ profile, related to the gradient of the electric field magnitude, is obtained:

$$\hat{G}^{(1)}(x) = \frac{2n\pi}{L}\sum_{n=1}^{N}\left[a_n \cos\left(\frac{2n\pi}{L}x\right) - b_n \sin\left(\frac{2n\pi}{L}x\right)\right] \quad \text{(S20)}$$

As the extracted data from COMSOL is a set of points, the integrals in eqns. (S12)-(S14) and (S16)-(S18) must be carried out numerically. For this, the *scipy* library was used in a Python code to apply the appropriate Simpson rule to the E and E^2 data. This same code calculated their Fourier coefficients and stored them to be used later in their analytic predictions. It should be noted that, in eqns. (S13) and (S14), the Fourier series is truncated on the N-th term, due to the discrete nature of the data. As frequency increases, the number of points needed to get a well-defined sinusoidal function increases as well. As it is hard to define a truncation term given the number of points of the raw data, an algorithm was implemented in the mentioned code to expand the distribution until the mean squared error is no longer minimized.

Because of the direct proportionality relation between E and the applied voltage in the channel (V), it is asserted that $\frac{E_1}{V_1} = \frac{E_2}{V_2}$. With this, the curve fitting of E can be performed for only one voltage input and then rescale it as necessary. Due to the properties of the analyzed channels, this process was performed for terminals A and C together and terminal B individually as terminals A and C provide an extra DC-bias signal and terminal B provides either a DC or AC+DC signal. For this study, a voltage $V_0 = 100$ V was used for terminal B and the distribution generated by terminals A and C was directly analyzed. Results of the performed curve fittings for both E and $|\nabla E^2|$ are illustrated in **Figure S4**.

With all this, the predicted particle velocity is defined as:

$$\hat{v}_p(t,x) = \left(\mu_{EO} + \mu_{EP}^{(1)}\right)\left[\hat{E}_{A,C}(x) + V_B(t)\hat{E}_B(x)\right] + \mu_{EP}^{(3)}\left[\hat{E}_{A,C}(x) + V_B(t)\hat{E}_B(x)\right]^3 + \mu_{DEP}\hat{H}(x,t) \quad \text{(S21)}$$

where:

$$\hat{H}(x,t) = \hat{G}_{A,C}^{(2)}(x) + V_B^2(t)\hat{G}_B^{(2)}(x) + 2V_B(t)\left[\hat{E}_{A,C}(x)\hat{G}_B^{(1)}(x) + \hat{E}_B(x)\hat{G}_{A,C}^{(1)}(x)\right] \quad \text{(S22)}$$

which is obtained when $\left|\nabla\left[\hat{E}_{A,C}(x) + V_B(t)\hat{E}_B(x)\right]^2\right|$ is expanded. Additionally:

$$V_B(t) = \frac{1}{V_0}\left[V_{DC} + V_p \sin(\omega t)\right] \quad \text{(S23)}$$

Finally, an estimation of retention time can be obtained by solving eqn. (S10), which was carried out with the use of the 4th order Runge-Kutta method implemented in the previously mentioned code and taking the temporal step as the one used in the function generators in the lab (0.06 s). Thus, $t_{R,p}$ is extracted when the condition $\hat{r}(t_{R,p}) \geq L$ is met.

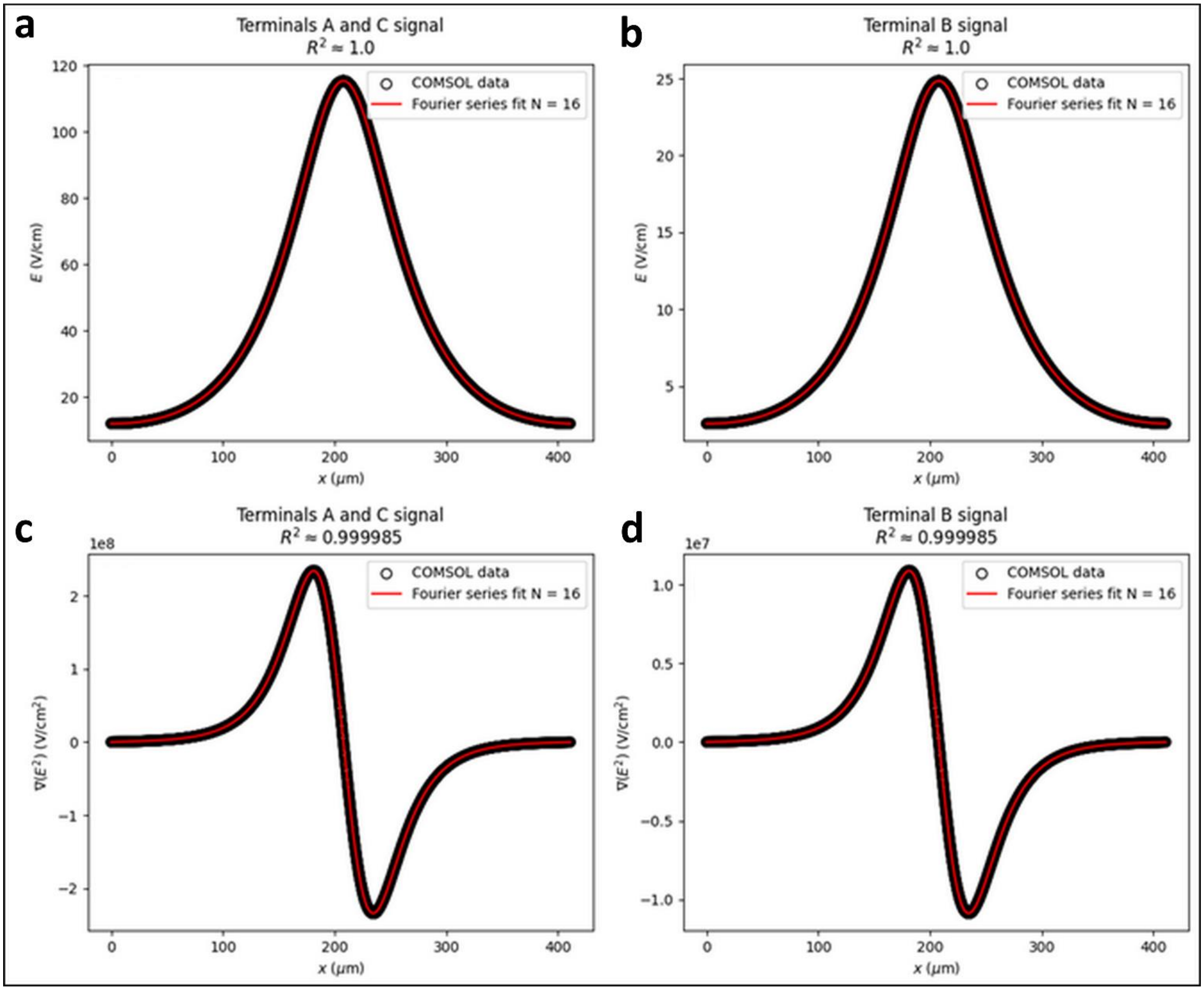

Figure S4. Results of curve fitting using a Fourier series expansion of the E-field profile. In scattered marking the E-field provided by COMSOL is plotted and the red line contains the curve fitting attained with the Fourier series expansion. For both data sets, the truncation element was found to be 16. The benchmark selected to define a good fit was the determination coefficient R^2 rounded to six decimals. **(A, C)** Contains the extracted data of the E-field and $\left|\nabla E^2\right|$ with their respective Fourier series expansions for terminals A and C, **(B, D)** contains the extracted data of the E-field and $\left|\nabla E^2\right|$ with their respective Fourier series expansions for terminal B.

5. Comparison of the velocity magnitudes of all four EK phenomena: includes Figure S5.

Depiction of overall and individual velocities over the cutline is shown in **Figure S5**. Based on the prediction of individual velocities resulting from each one of the four electrokinetic phenomena, it can be observed that for all separations (Separation IDs 1-3), the dominant EK phenomena contributing to the overall cell velocities is electrophoresis. It is also important to note that at the selected DC-biased AC voltage (**Table 2** in the manuscript), the magnitude of the nonlinear electrophoretic effects ($\mathrm{EP}_{\mathrm{NL}}^{(3)}$) was kept low/moderate for the first eluting cell species and low/high for the second eluting cell species in such a way that the discrimination between cell species is enhanced, and, allowing the separations to take place by exploiting electrophoretic effects.

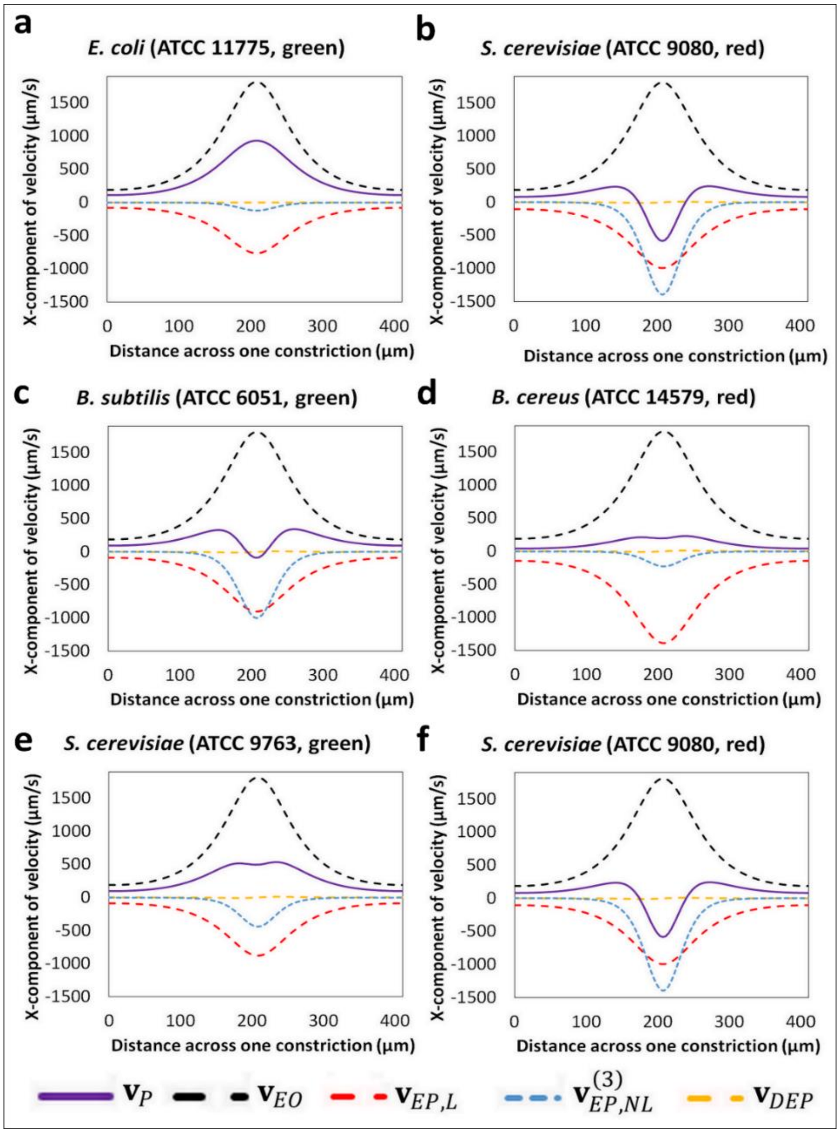

Figure S5. Predicted overall and individual cell velocities exerted by the four EK phenomena across the cutline (**Figure S3**), for all the cell separations (Separation IDs 1-3). (**A,B**) Separation ID 1 for *E. coli* (ATCC 11775, green) and *S. cerevisiae* (ATCC 9080, red) cells from prokaryotic and eukaryotic domains respectively. (**C, D**) Separation ID 2 for *B. subtilis* (ATCC 6051, green) and *B. cereus* (ATCC 14579, red) cells, respectively, from the same prokaryotic domain and different species. (**E,F**) Separation ID 3 for *S. cerevisiae* (ATCC 9763, green) and *S. cerevisiae* (ATCC 9080, red) cells, respectively, from the same eukaryotic domain, same species and different microbial strains.

6. Reproducibility between repetitions: includes Table S4 and Figure S6

Table S4. Values of retention times for three distinct experimental repetitions of each separation.

Separation ID	Cell ID – label color	$t_{R,e}$ Repetition 1 (s)	$t_{R,e}$ Repetition 2 (s)	$t_{R,e}$ Repetition 3 (s)	$t_{R,e}$ Average (s)	Range of stdev (%)
1	*E. coli* (ATCC 11775) - Green	301.0	300.0	290.0	297.0	2.1
	S. cerevisiae (ATCC 9080) - Red	489.0	470.0	509.0	489.3	3.3
2	*B. subtilis* (ATCC 6051) - Green	340.0	321.0	330.0	330.3	2.9
	B. cereus (ATCC 14579) - Red	606.0	670.0	626.0	634.0	5.2
3	*S. cerevisiae* (ATCC 9763) - Green	352.0	348.0	328.0	342.7	3.1
	S. cerevisiae (ATCC 9080) - Red	465.0	447.0	456.0	456.0	2.1

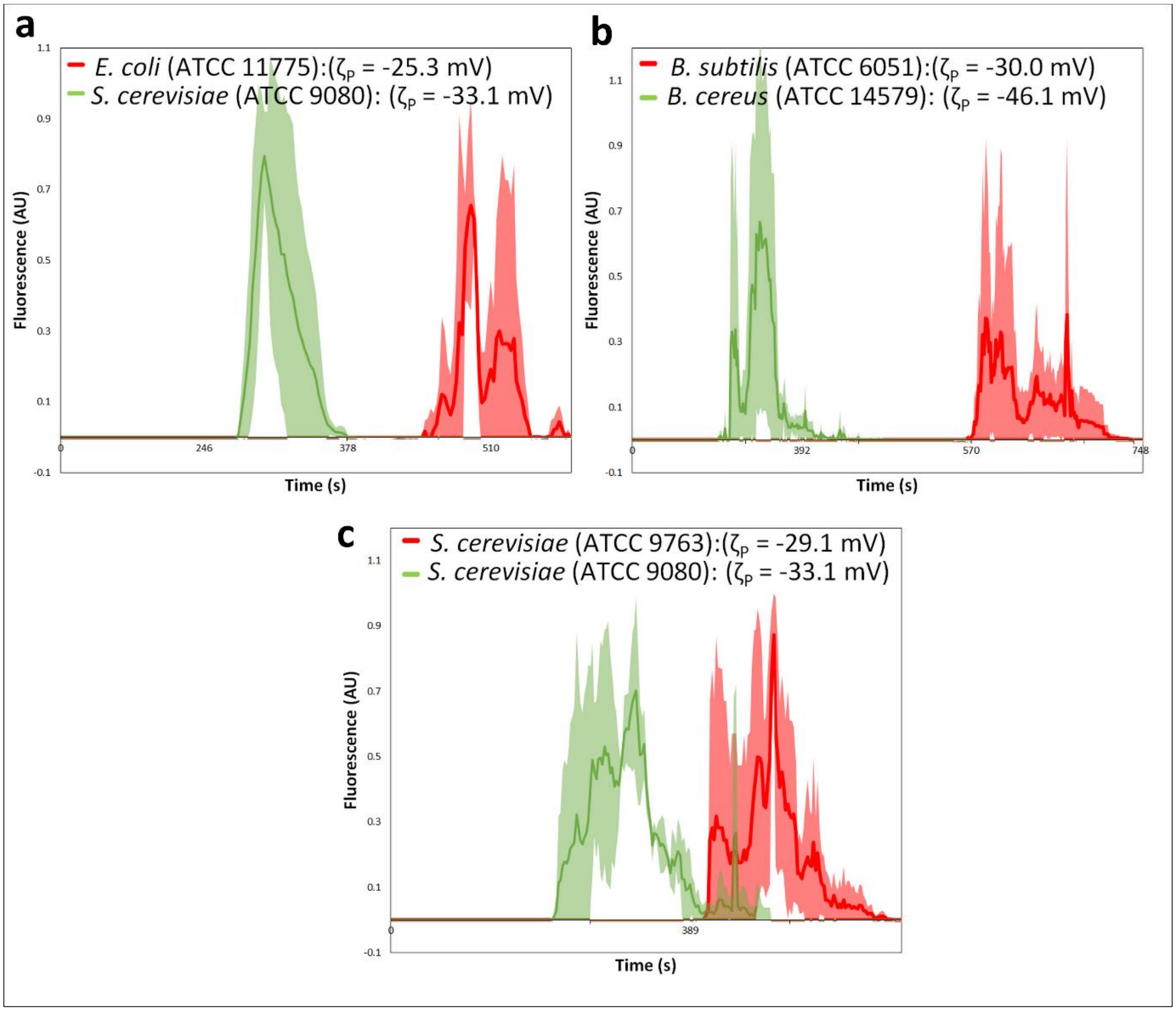

Figure S6. Confidence interval plots of the electropherograms indicating reproducibility between experiments for all the cell separations (Separation IDs 1-3). (**A**) Separation ID 1 for *E. coli* (ATCC 11775, green) and *S. cerevisiae* (ATCC 9080, red) cells. (**B**) Separation ID 2 for *B. subtilis* (ATCC 6051, green) and *B. cereus* (ATCC 14579, red) cells. (**C**) Separation ID 3 for *S. cerevisiae* (ATCC 9763, green) and *S. cerevisiae* (ATCC 9080, red) cells.

7. References

1. Cobos, R.; Khair, A.S. Nonlinear Electrophoretic Velocity of a Spherical Colloidal Particle. *J. Fluid Mech.* **2023**, *968*, A14, doi:10.1017/jfm.2023.537.
2. Schnitzer, O.; Zeyde, R.; Yavneh, I.; Yariv, E. Weakly Nonlinear Electrophoresis of a Highly Charged Colloidal Particle. *Phys. Fluids* **2013**, *25*, 052004, doi:10.1063/1.4804672.
3. Schnitzer, O.; Yariv, E. Nonlinear Electrophoresis at Arbitrary Field Strengths: Small-Dukhin-Number Analysis. *Phys. Fluids* **2014**, *26*, 122002, doi:10.1063/1.4902331.
4. Gallo-Villanueva, R.C.; Sano, M.B.; Lapizco-Encinas, B.H.; Davalos, R.V. Joule Heating Effects on Particle Immobilization in Insulator-Based Dielectrophoretic Devices. Electrophoresis 2014, 35, 352–361. https://doi.org/10.1002/elps.201300171.
5. Neter, J.; Wasserman, W.; Kutner, M.H. Applied Linear Regression Models; Richard D Irwin, Inc.: Homewood, Illinois, USA, 2004.
